# NEIGHBORHOOD SOCIAL CAPITAL IS ASSOCIATED WITH VARIABILITY IN AMBULATORY COGNITIVE PERFORMANCE

**DOI:** 10.1093/geroni/igae098.2656

**Published:** 2024-12-31

**Authors:** Jinshil Hyun, Eric Cerino, Mindy Katz, Richard Lipton, Martin Sliwinski

**Affiliations:** Albert Einstein College of Medicine, Bronx, New York, United States; Northern Arizona University, Flagstaff, Arizona, United States; Albert Einstein College of Medicine, Bronx, New York, United States; Albert Einstein College of Medicine, Bronx, New York, United States; Pennsylvania State University, University Park, Pennsylvania, United States

## Abstract

Prior studies suggested that within-person variability in cognitive task performance may be an early indicator of normative and pathologic cognitive aging. However, the influence of upstream social determinants of health (e.g. neighborhood) on cognitive variability is not well characterized. The aim of this study is to examine whether neighborhood social capital is associated with within-person variability in various cognitive domains and whether these associations are mediated by individual-level behavioral factors (i.e., physical activity, social interaction). The analysis sample included 316 individuals in Bronx, NY, enrolled in the Einstein Aging Study (mean age=77). Participants performed brief tests of processing speed, memory binding, and working memory on a smartphone up to six times daily over a two-week period. Neighborhood social capital was operationalized as a composite variable comprising economic connectedness, clustering, and volunteering rates, linked to participants’ address through zip code tabulation area. Results from heterogeneous variance multilevel models suggested that living in areas with lower levels of social capital was significantly associated with greater variability in processing speed and memory binding (ps<.001). Controlling for individual-level physical activity reduced the magnitude of the effects of neighborhood social capital, suggesting potential (and partial) mediating role of physical activity. In conclusion, living in areas with better community social capital may reduce the risks of cognitive impairment. It would be crucial to support upstream neighborhood-level interventions to prevent cognitive impairment among older adults. Future research should examine more precise behavioral mechanisms linked to neighborhood environments to better suggest effective intervention strategies to promote cognitive health.

